# Cellular Stress Responses in Radiotherapy

**DOI:** 10.3390/cells8091105

**Published:** 2019-09-18

**Authors:** Wanyeon Kim, Sungmin Lee, Danbi Seo, Dain Kim, Kyeongmin Kim, EunGi Kim, JiHoon Kang, Ki Moon Seong, HyeSook Youn, BuHyun Youn

**Affiliations:** 1 Department of Biology Education, Korea National University of Education, Cheongju-si, Chungbuk 28173, Korea; wykim82@knue.ac.kr; 2 Department of Science Education, Korea National University of Education, Cheongju-si, Chungbuk 28173, Korea; dbseo130@gmail.com (D.S.); ekdls5371@gmail.com (D.K.); 3 Department of Integrated Biological Science, Pusan National University, Busan 46241, Korea; smlee1048@gmail.com (S.L.); minnnny@gmail.com (K.K.); eungikim89@gmail.com (E.K.); jhkang4293@gmail.com (J.K.); 4 Laboratory of Low Dose Risk Assessment, National Radiation Emergency Medical Center, Korea Institute of Radiological & Medical Sciences, Seoul 01812, Korea; skmhanul@kirams.re.kr; 5 Department of Integrative Bioscience and Biotechnology, Sejong University, Seoul 05006, Korea; 6 Department of Biological Sciences, Pusan National University, Busan 46241, Korea

**Keywords:** radiation response, radioresistance, reactive oxygen species, DNA damage response, lipid peroxidation, mitochondrial damage, ER stress, autophagy

## Abstract

Radiotherapy is one of the major cancer treatment strategies. Exposure to penetrating radiation causes cellular stress, directly or indirectly, due to the generation of reactive oxygen species, DNA damage, and subcellular organelle damage and autophagy. These radiation-induced damage responses cooperatively contribute to cancer cell death, but paradoxically, radiotherapy also causes the activation of damage-repair and survival signaling to alleviate radiation-induced cytotoxic effects in a small percentage of cancer cells, and these activations are responsible for tumor radio-resistance. The present study describes the molecular mechanisms responsible for radiation-induced cellular stress response and radioresistance, and the therapeutic approaches used to overcome radioresistance.

## 1. Introduction

Radiotherapy is a powerful tool in the fight against cancer. Nowadays, approximately 50% of all cancer patients are administered radiotherapy plus surgery, and/or chemotherapy [1]. Many efforts have been expended in improving the efficacies of radiotherapy, and as a result, therapeutic outcomes have improved, and the incidences of side effects associated with damage to nearby normal tissues have been reduced. Nevertheless, radiotherapeutic efficacy is limited by the development of radioresistance and by damage to normal tissues [2,3,4]. Radiotherapy potently induces massive cell death by triggering the activation of death signaling in cancer cells via the generation of reactive oxygen species (ROS), DNA damage, and stress response in subcellular organelles, such as endoplasmic reticulum (ER) and mitochondria [4,5,6,7]. However, a small portion of cancer cells may survive by activating compensatory survival signaling involving, for example, damage-repair signaling (e.g., ROS scavenging), DNA repair, unfolded protein response (UPR), and the induction of autophagy [8,9,10]. Cancer cells that survive radiotherapy exhibit radioresistance and are able to promote tumor regrowth and tumor recurrence, characterized by aggressive disease development [11,12,13]. Since radioresistance is a major cause of therapy failure, understanding signaling response in tumor cells that are exposed to radiation is essential for improving radiotherapeutic efficacies. In addition, further studies are required to increase the radiosensitivities of tumor cells [14]. The present study aims to provide a review of intracellular stress response during radiotherapy, as well as the contributions it makes to radiation-induced cell death and/or the possible occurrence of radioresistance. We also provide a brief review of recent clinical approaches used to promote tumor radiosensitization.

## 2. Radiation-Induced ROS Response

ROS have been shown to play important roles during cell proliferation, cell motility, the cell cycle, and apoptosis [15,16]. During radiotherapy, ROS, including the superoxide anion (O_2_^−^), hydroxyl radicals (OH^−^) and hydrogen peroxide (H_2_O_2_), are generated by the radiolysis of water in extracellular environments, and these highly reactive entities are toxic to tumor cells and nearby normal tissues [17]. In addition, radiation can induce endogenous ROS production in mitochondria [18], and alter mitochondrial membrane permeability, which in turn, further stimulates ROS production [19,20]. Excessive levels of ROS can also disrupt components of the electron transport chain in mitochondria, induce intracellular redox system imbalances [21], and cause oxidative stress by reacting with biological molecules such as lipids, proteins, and DNA to cause lipid peroxidation, protein misfolding, and DNA strand breaks. On the other hand, endogenous antioxidant systems protect against radiation-induced oxidative stress by scavenging free radicals. For example, O_2_^-^ can be converted to H_2_O_2_ by superoxide dismutases (SODs), and catalase and peroxidases can convert H_2_O_2_ to water and O_2_ [22,23].

**Figure 1:**
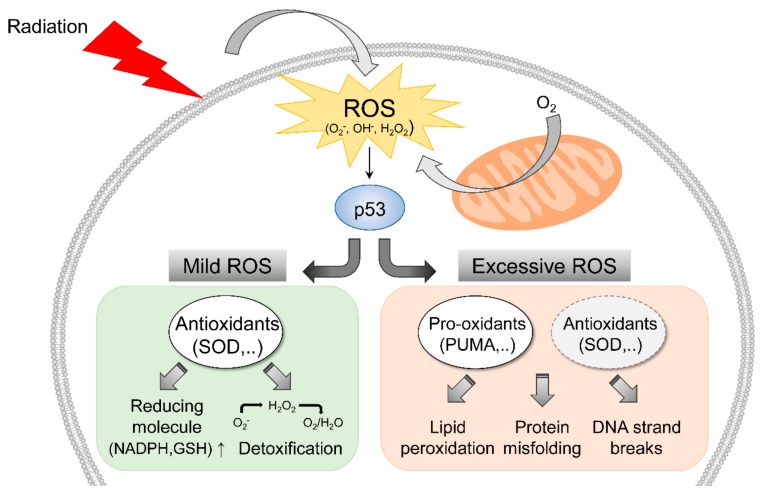
Radiation-induced reactive oxygen species (ROS) response associated with p53 signaling. Irradiation increases intracellular ROS levels facilitated by radiation-mediated mitochondrial damage. In the presence of elevated ROS levels, p53 may importantly ameliorate radiation-induced oxidative stress.

During the response to radiation-induced oxidative stress, p53 may play a pivotal role in the regulation of redox status (Figure 1). When intracellular ROS levels are relatively low, activated p53 can promote the transcriptions of antioxidant enzymes, such as manganese SOD, glutathione peroxidase 1, members of the sestrin gene family, and glutaminase 2 [24,25,26], and these genes participate in the detoxification of various ROS and upregulate reducing molecules, such as nicotinamide adenine dinucleotide phosphate (NADPH) and glutathione. However, when intracellular ROS levels are extensively increased by radiation, p53 can be activated by JNK signaling, which is responsible for the upregulations of pro-oxidant genes, like p53-upregulated modulator of apoptosis (PUMA), p67phox, and p53-inducible genes [27,28,29,30]. PUMA can promote ROS production by altering mitochondrial permeability, which is associated with p53-dependent apoptosis. p67phox (encoded by the neutrophil cytosol factor 2 gene) is a subunit of NADPH oxidase complex and may play a critical role in the escalation of cytosolic O_2_^−^ levels. Moreover, it has been reported that p53 might be involved in the suppression of antioxidants associated with nuclear factor-E2-related factor (Nrf2) [31], which is capable of inducing the transcriptions of antioxidant genes by binding to antioxidant response element (ARE) in their promoters regions. p53 can directly block these ARE sites, and thus, suppress Nrf2-mediated transcription. These interactions indicate that high levels of ROS accumulation stabilize p53 protein and render cells liable to apoptosis induction. Thus, the dual functions of p53 probably contribute to cell fate decision-making in response to low or high levels of intracellular ROS. In oncology, radiation exposure can be a potent option that enhances intracellular ROS levels and induces tumor cell death in a p53-dependent manner.

Tumor cells can adapt to radiation-induced oxidative stress mediated in various ways, for example, by increasing antioxidant levels, altering metabolism, and generating hypoxia response. In particular, intratumoral hypoxia, which is caused by an inadequate vascular system and tumor growth, is responsible for the suppression of apoptosis during radiotherapy as low oxygen availability limits ROS generation [32]. Paradoxically, radiation can disrupt in vivo vascular systems around tumors, induce hypoxia response, and activate hypoxia-inducible factor 1 (HIF1) in cancer cells, and thus, reduce the generation of intratumoral ROS [33]. Several molecular studies have explained the roles played by ROS during HIF1 activation [34]. Understanding the role of HIF1 in ROS response is important because tumoral HIF1 activation is not observed in normal cells. It has been widely reported that HIF1 increases the expression of vascular endothelial growth factor (VEGF), which, in the normal state, promotes angiogenesis. However, VEGF stimulation in tumors leads to abnormal tumor angiogenesis that prevents the homogeneous distribution of blood and acts to suppress ROS generation [35]. HIF1 can also allow cancer cells to rely on oxygen consumption by activating glycolytic metabolism, and inhibiting mitochondrial oxidative phosphorylation against low oxygen availability, by upregulating pyruvate dehydrogenase kinase 1 (PDK1), which suppresses respiratory ROS generation [36]. HIF1 can also induce the expression of anti-oxidants, and thus, alleviate ROS-induced oxidative damage in cancer cells [37]. As described above, hypoxia and radiation can both stabilize and activate HIF1, and intratumoral HIF1 plays a leading role in the suppression of ROS. Furthermore, HIF1 activation has cytoprotective effects on cancer cells during radiotherapy and contributes to tumor radioresistance and the growth of surviving cells, the microenvironmental control of hypoxia by reoxygenation, and the targeting of HIF1 offer potent strategies for successful radiotherapy.

## 3. Radiation-Induced DNA Damage Response

Radiation can damage DNA directly by ionization, and indirectly by ROS generation, and thus, induce single-strand breaks (SSBs), base oxidation, apurinic, or apyrimidinic (AP) sites, and most importantly, double-strand breaks (DSBs). Typically, the beneficial outcomes of radiotherapy may be achieved by DNA double-strand breaks caused by high energy damage to DNA backbones. ROS also promote the oxidations of DNA bases, which can be recognized by base excision repair (BER) systems. Oxidized bases are rapidly produced by irradiation and overload BER pathways, and thus, increase the likelihood of DSB generation. In this manner, mitochondrial and nuclear DNA may be damaged sufficiently to result in tumor cell death. Although non-DSB damage, including SSBs, base lesions, and AP sites are more frequently generated by irradiation, these damage regions can be repaired with high accuracy and fidelity by various repair systems [38]. For the repair of base lesions, DNA glycosylases can bind to and cleave a damaged base, leading to an abasic site. The abasic site can be recognized and excised by AP endonucleases and the gap is further repaired by the interaction with DNA polymerases, DNA ligases, and X-ray repair cross-complementing protein 1 (XRCC1) [39,40,41]. In addition, poly(ADP-ribose) polymerase 1 (PARP1) may recognize SSBs and lead to poly(ADP) ribosylation for the recruitment of other SSB-repair proteins, including AP endonuclease 1 (APE1), flap endonuclease 1, proliferating cell nuclear antigen (PCNA), DNA polymerases, and XRCC1 [42,43,44].

**Figure 2:**
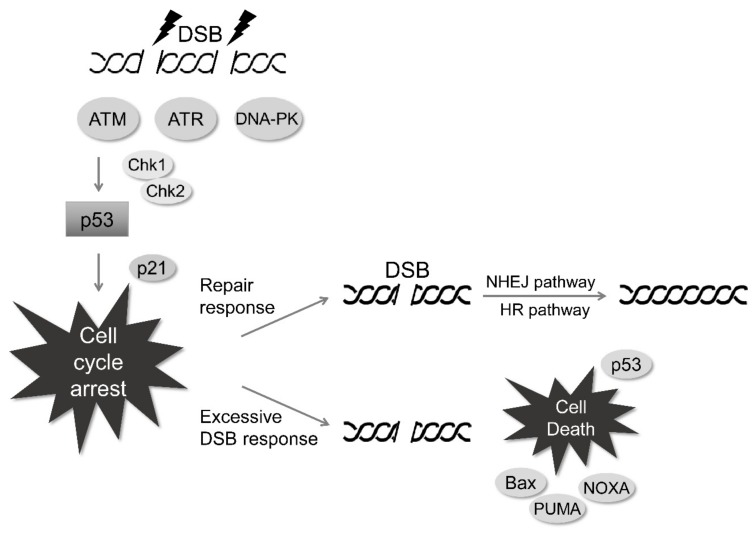
Radiation-induced double-strand breaks (DSB) response. When DSBs are induced by irradiation, DNA damage-sensing and repair proteins such as ATM, ATR, DNA-PK, H2AX, MDC1, Chk1, and Chk2 are activated. Subsequently, p53 is activated and induces cell cycle arrest for the homologous recombination (HR) or non-homologous end joining (NHEJ) pathways or induces apoptosis by upregulating proapoptotic genes.

However, radiation can lead to complex DNA damage, with multiple non-DSB lesions, termed clustered DNA damage [45,46,47]. In particular, the clustered DNA damage, in which more than two SSBs are formed nearly on both DNA strands, may be recognized as a type of DSBs. Since the clustered DNA damage may require prolonged activation of repair systems, it is associated with incomplete repair of damaged sites, and an increase in mutation rates, responsible for radiation-induced genomic instability. Thus, the clustered DNA damage and DSBs induced by radiation may cooperatively contribute to cell death. When DSBs are generated by exposure to radiation, several sensor proteins, such as ataxia-telangiectasia mutated (ATM), ataxia-telangiectasia and Rad3-related (ATR), and DNA-dependent protein kinase (DNA-PK) are initially activated to recruit downstream proteins in the DNA damage response pathway (Figure 2) [48]. Radiation-induced phosphorylations of H2AX (γH2AX, a substrate of ATM), ATR and DNA-PK and the resultant recruitment of mediator of DNA damage checkpoint protein 1 (MDC1) in DNA-damaged foci, and MDC1 might facilitate the recruitment of DNA damage response proteins such as ring finger protein 8 (RNF8), RNF168, tumor suppressor p53-binding protein 1 (53BP1), breast cancer type 1 susceptibility protein 1 (BRCA1) and BRCA2 [49,50,51]. Furthermore, these responses might result in the phosphorylation of checkpoint kinase 1 (Chk1) and Chk2, which may also be directly activated by ATM/ATR to stabilize and activate p53 during irradiation. Activated p53 participates in p21-associated cell cycle arrest [52], and after cell cycle arrest, break sites are repaired by either the non-homologous end joining (NHEJ) pathway involving Ku70/Ku80 dimer, XRCC4, and XRCC4-like factor, or by the homologous recombination (HR) pathway, involving MRE11/RAD50/NBS1 complex. Based on the molecular characteristics of NHEJ, the DSB repair may have potential shortcomings, accompanied by incomplete repair and high mutation rates, contributing to radiation-induced cell death. Typically, normal cells can trigger cell cycle arrest in response to irradiation via the activation of cell cycle checkpoints, at least in part, including p53-dependent p21 activation and subsequent inhibition of cyclin-dependent kinase 4 (Cdk4)/cyclin D and Cdk2/cyclin E complexes. It may also allow normal cells to have a great opportunity to retain the sufficient time for DNA damage repair [53,54]. In tumor cells possessing genetic defects in sensor or repair proteins, incomplete DNA repair would trigger cell death signaling associated with the p53-dependent expressions of proapoptotic proteins such as PUMA, phorbol-12-myristate-13-acetate-induced protein 1 (also known as NOXA) and Bcl2-associated X protein (Bax) [55,56,57]. Supportive studies have reported that the knockdown of MDC1 results in impaired ATM signaling and defective DNA repair, and consequently enhances radiation sensitivity [58,59]. However, regarding tumor cells, the Oncomine and the Cancer Genome Atlas Program (TCGA) databases show that the expressions of DNA repair genes (e.g., *BRCA1*, *BRCA2*, *ATM*, *PRKDC* (gene name for DNA-PK), and *MDC1*) are upregulated in most tumor types, and that, 10–20% of tumor cells show mutations in these genes, which makes them resistant to DNA damage and radiotherapy-induced apoptosis. In addition, enhanced transcription in tumor cells leads to severe genomic instability, which represents a molecular basis for therapeutic resistance and further tumor development [60]. Interestingly, the expressions of some DNA repair proteins (e.g., RNF8, RNF168, and 53BP1) were reported to be similar in tumor and normal cells. Further studies on DNA damage response are needed to identify the molecular mechanisms in tumor cells, that are responsible for maintaining the balance between DNA damage response and genomic instability.

## 4. Radiation-Induced Subcellular Organelle Response

### 4.1. Membrane-Associated Signaling in Response to Irradiation

**Figure 3:**
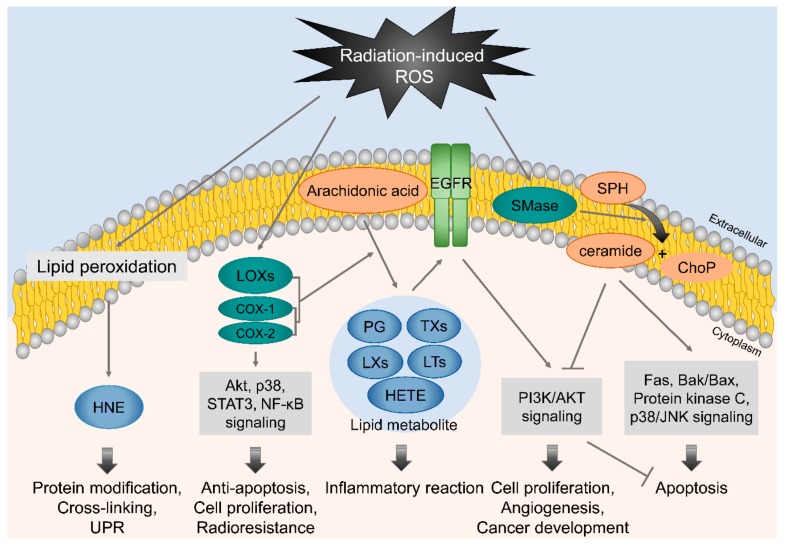
Radiation-induced lipid peroxidation and ceramide signaling. Exposure of the plasma membrane to penetrating radiation leads to the production of homologous recombination (HNE), arachidonic acid-derived lipid metabolites, and ceramide. HNE is associated with the stimulation of unfolded protein response (UPR), and arachidonic acid metabolites promote cell proliferation, inflammation, and protect cells from apoptosis, and thus, contribute to tumor radioresistance. On the other hand, ceramide triggers apoptosis by activating Fas and Bak/Bax signaling and inhibiting PI3K/Akt signaling.

The plasma membrane lipid bilayer is exposed to radiation and radiation-induced ROS, and thus, results in lipid peroxidation, including the peroxidation of polyunsaturated fatty acids (PUFAs) (Figure 3). Lipid oxidative damage is associated with plasma membrane permeability and membrane protein and molecular transport disruptions [61,62]. After radiation exposure, PUFAs can be converted to various lipid peroxide derivatives, such as isoprostanes, malondialdehyde, and lipid hydroperoxides. 4-Hydroxy-2-nonenal (HNE), produced by lipid peroxidation, reacts easily with amino or thiol groups and modifies and cross-links proteins, including oxidoreductases, transferases, and kinases [63]. High HNE levels can also trigger unfolded protein response (UPR) through pathways involving protein kinase R (PKR)-like endoplasmic reticulum kinase (PERK). This activates the transcription factor 6 (ATF6) and inositol requirement 1 (IRE1), accompanied by JNK and p38 signaling, which suggests that HNE might act as an upstream modulator between radiation-induced ROS response and ER stress [64]. Moreover, ROS can trigger the activation of sphingomyelinase, which catalyzes sphingomyelin hydrolysis in plasma membranes, and thus, induces ceramide production [65]. Ceramide can be produced by ceramide synthase, which is activated by radiation-induced DSBs [66], and is associated with both the extrinsic and intrinsic apoptotic pathways via the activation of Fas signaling, Bak/Bax signaling, protein kinase C (PKC) signaling, p38/JNK signaling, or Ca^2+^ signaling, or via the inhibition of Akt signaling.

Irradiation can also cause the activation of cyclooxygenases (COXs) and lipoxygenases (LOXs), which contribute to the production of bioactive lipid metabolites from arachidonic acids in plasma membranes [67,68,69]. These lipid metabolites, including prostaglandins, thromboxanes, lipoxins, leukotrienes, and hydroxyeicosatetraenoic acids, which are mainly involved in inflammatory response. Although, they are also associated with cell proliferation, angiogenesis, and cancer development via the activations of EGFR signaling and PI3K/ATK signaling. In addition to the production of lipid metabolites, COX-2 can be overexpressed by irradiation and has anti-apoptotic and cell proliferation promoting effects, which are associated with Akt, p38, STAT3, and NF-κB signaling, and thus, contributes to radioresistance in cancer cells [70,71,72,73]. Since the activations of COXs and LOXs and their lipid metabolites are mainly associated with radioresistance and cancer development, the pharmacological inhibitions of COXs and LOXs, combined with radiotherapy, offer a promising option for enhancing therapeutic efficacy.

### 4.2. Mitochondrial Damage Induced by Radiation

**Figure 4:**
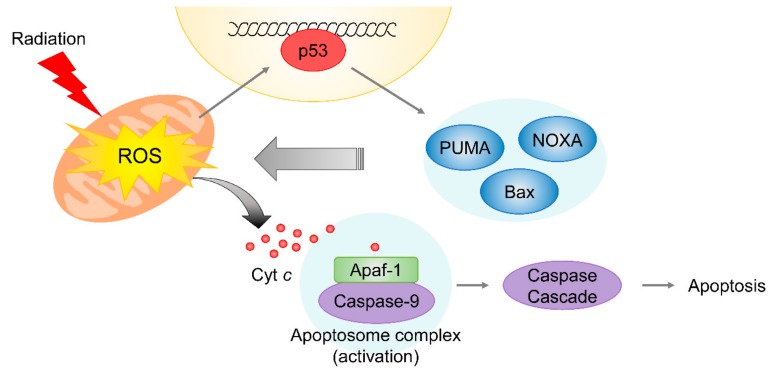
Radiation-induced mitochondrial response. Radiation induces mitochondrial damage largely via ROS generation. Excessive ROS levels and radiation-induced p53-dependent upregulations of PUMA and Bak/Bax result in mitochondrial membrane permeabilization and subsequent release of cytochrome *c* into cytosol, and thus, promote intrinsic apoptotic signaling.

As mentioned above, radiation-induced mitochondrial damage is accompanied by ROS generation during radiotherapy [74]. In mitochondria, ROS are produced by the tricarboxylic acid (TCA) cycle and the electron transport chain during aerobic respiration. Since repair systems are not well-developed in mitochondria, excessive ROS generation by irradiation and endogenous ROS contribute to extensive and long-term mitochondrial DNA (mtDNA) damage [75]. The damage might be linked to mitochondrial genomic instability and permanent mitochondrial malfunction, leading to stimulation of the intrinsic apoptotic pathway, which involves cytochrome *c* release. In addition, the delayed activation of p53 results in the expressions of pro-apoptotic proteins, including PUMA, NOXA, and Bax [55,56,57], which are also involved in mitochondrial membrane permeabilization and subsequent cytochrome *c* release. Released cytochrome *c* in cytosol triggers the intrinsic apoptotic signaling via the formation of apoptosome complex, consisting of cytochrome *c*, apoptotic protease activating factor 1, and caspase 9, which subsequently stimulates caspase 3/7 cascade activation (Figure 4) [76].

Mitochondrial statuses differ in tumor cells and normal cells and manifests as different mitochondrial stress responses to irradiation. Despite the importance of mitochondrial oxidative phosphorylation in energy metabolism, mitochondrial dysfunction has been implicated in cancer cells associated with the Warburg effect. Tumor cells adapting to hypoxic conditions favor aerobic glycolysis, while radiation-induced HIF1 stabilization might be further accompanied by mitochondrial defects that contribute to a glycolytic phenotype via the upregulations of glycolytic enzymes and the suppression of TCA cycle entry [36,77,78,79,80]. Glycolytic reprogramming increases the glucose uptake and accelerates the productions of pentose phosphate pathway intermediates, such as glucose-6-phosphate, fructose-6-phosphate, and glyceraldehyde-3-phosphate [81,82,83]. Furthermore, increased biogenesis of nucleotides and amino acids, from the pentose phosphate pathway, could provide building blocks for cancer cell proliferation. In addition, several antioxidants, such as NADPH and glutathione could be produced and the antioxidant capacity enhanced as a result, which would contribute to cancer cell radioresistance. HIF1 is responsible for inducing autophagy for mitochondrial degradation and providing building blocks for cell survival [84]. Thus, tumor cells create a more protective intracellular environment by glycolytic reprogramming, and the presence of mitochondrial defects, accompanied by the adaptation to hypoxic conditions, provide radioresistant properties, as well as survival and growth benefits.

### 4.3. Endoplasmic Reticulum Stress in Response to Radiation

**Figure 5:**
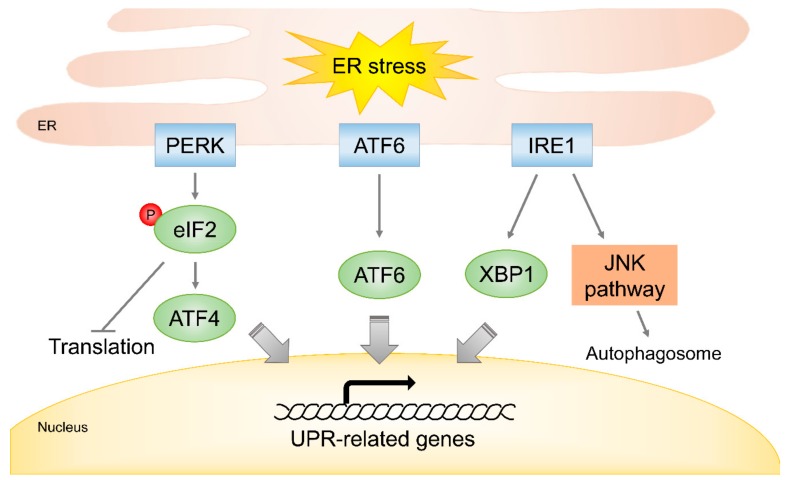
Radiation-induced ER stress response. Radiation can induce ER stress directly or indirectly by generating ROS. Under radiation-induced ER stress, specific signaling by PERK, ATF6, and IRE1 may be activated, and augment the upregulations of UPR-related genes to improve chaperone activity and induce autophagy to recover and recycle misfolded proteins.

In ER, subcellular organelles, that are responsible for the synthesis and folding of membrane proteins and for calcium ion storage, can sensitively respond to external and internal stimuli, like irradiation and ROS. The functions of ER are disrupted under cellular stress and trigger specific signals using PERK, ATF6, and IRE1 [85]. These proteins are responsible for UPR, as well as alleviating misfolded protein accumulations and regulate global translation under ER stress, but excessive activation of UPR signaling is linked with the inductions of autophagy or apoptosis (Figure 5). The activation of PERK results in the inhibition of global translation through the phosphorylation of eukaryotic translation initiation factor α subunit (eIF2α), and the phosphorylated eIF2α is able to contribute to the stimulation of apoptosis by inducing the expressions of growth arrest and DNA damage 34 (GADD34), GADD153, and CCAAT/enhancer binding protein homologous protein (CHOP) [86,87,88]. ATF6 binds to the promoters of UPR-related genes, including *Grp78*, *Grp94*, *CHOP*, *X-box binding protein 1* (*XBP1*), and several chaperones [89,90]. IRE1 aids XBP1 activation by contributing to the upregulations of UPR genes. In addition, IRE1 may be involved in JNK signaling to facilitate autophagosome formation under ER stress [91]. Furthermore, it has been reported that the over-expressions of CHOP and GADD153 are correlated with increased ER stress sensitivity, as well as ROS levels and reduced GSH levels [92].

The induction of ER stress response might contribute to adaptive survival signaling in cancer cells during radiotherapy. It was observed that the global expressions of ER stress-responsive genes, such as *PERK*, *ATF4*, *ATF6*, *GADD34*, and *IRE1* were increased by the irradiation of glioblastoma cells [93]. In particular, ATF6 activated by irradiation was associated with the upregulation of Notch1, which is not directly involved in UPR, but plays a pivotal role in cell proliferation and protection from apoptosis, and thus, contributes to the radioresistance of glioblastoma cells [93,94]. Other studies have reported that cells exposed to radiation exhibit the activation of PERK-induced eIF2α and ATF4 signaling, as well as enhancing UPR gene expressions (e.g., *BiP*, *Grp94*, and *XBP1*) [95,96]. In addition, UPR-independent signaling pathways mediated by PERK, IRE1, and ATF6 might be responsible for enhanced tumor growth and angiogenesis through the transcriptional regulations of VEGF, fibroblast growth factor 2, connective tissue growth factor, and interleukin 6 [97,98]. These results indicate that the upregulations of ER stress-associated genes, in cancer cells, are intimately involved in radioresistance and cell survival, and suggest targeting these genes might enhance tumor radiosensitivity.

## 5. Radiation-Induced Autophagy

Autophagy is a process of metabolic recycling, and involves the self-digestion of subcellular organelles and molecules associated with lysosomes [99]. The metabolites recycled by lysosomal degradation serve as energy sources and building blocks, and contribute to cell survival under conditions of nutrient depletion. The autophagy pathway is initiated by ULK complex, which consists of UNC51-like kinase 1 (ULK1), autophagy-related protein 13 (ATG13), ATG101, and focal adhesion kinase family-interacting protein of 200 kDa (FIP200) signaling. Autophagy is capable of enabling cancer cells to survive, and maintains cell integrity by eliminating free radicals and damaged organelles [100]. It has been reported that the inhibition of autophagy-involving genes (e.g., *BECN1* and *ATGs*) results in the induction of apoptosis and is responsible for tumor radiosensitization [101]. HIF1 overexpression in response to irradiation may also be associated with autophagy induction, through the dissociation of Beclin-1/Bcl2 complex and the subsequent activation of Beclin-1 [102,103]. Thus, autophagy may provide an opportunity for cancer cells to survive in response to radiotherapy [104,105]. Nevertheless, recent evidence suggests that irradiation-induced cell death might be involved in autophagy (called autophagic cell death), which is morphologically distinguished from apoptosis [106,107]. The activation of autophagy has been reported to result in the radiosensitization and cell death of glioblastoma cells [108]. It has also been demonstrated that, p53 and damage-regulated autophagy modulator (DRAM, an effector protein of p53), are involved in radiation-induced autophagic breast cancer cell death [109]. In another study, it was suggested that autophagy might be an alternative mechanism of radiation-induced cell death in cancer cells with apoptotic pathway defects [110].

Cytoprotective autophagy in cancer cells limits radiotherapeutic efficacy. One of the major roles of autophagy is the removal and recycling of radiation-damaged intracellular organelles and molecules, which implies that autophagy provides cancer an opportunity to survive radiation-induced damage. In one study, autophagy mediated by Wnt3a-mediated signaling was found to provide radioresistance in squamous cell carcinoma of the head and neck [111], and in another, the radiosensitivity of colorectal cancer cells was enhanced by inhibiting ATG12-mediated autophagy using miR-214 [112]. These observations indicate that autophagy initiates cytoprotective signaling and reduces cancer cell radiosensitivity. During radiotherapy, it would appear that higher therapeutic efficacy would be achieved by activating autophagic cell death rather than cytoprotective autophagy.

## 6. Clinical Approaches to Radiosensitization Based on the Regulation of Cell Stress Responses

Many efforts have been made to identify pharmacological targets and to develop potent radiosensitizers that enhance tumor radiosensitivity. In this context, increased ROS production and inhibition of the antioxidant system provide potent options. Traditional cancer chemotherapeutic agents, such as cisplatin, bleomycin, and anthracyclines cause excessive ROS production and DNA damage, and lead to cancer cell death [113]. Daunorubicin, an anthracycline derivative, is used to treat acute myeloid leukemia, acute lymphocytic leukemia, and chronic myelogenous leukemia, and generates free-radicals, by interacting with cytochrome P450 reductase, which leads to ceramide-mediated apoptosis [114]. It has been proposed that high pharmacologic doses of ascorbic acid act as a H_2_O_2_-producing pro-oxidant that enhances cancer cell death, and treatment with ascorbic acid. This is carried out by the intravenous infusion as pharmacologic ascorbate in clinical trials on pancreatic cancer, which was found to result in radiosensitization of pancreatic cancer via H_2_O_2_-mediated oxidative stress with acceptable tolerability [115,116]. In addition, combinational treatment with motexafin gadolinium (an inhibitor of thioredoxin reductase and ribonucleotide reductase that leads to exhaustion of ROS scavenging capacity) and prompt whole brain radiotherapy produced a positive outcome by suppressing the metastatic conversion of non-small cell lung cancer to brain in a phase III trial [117].

The generation of bioactive lipid metabolites from peroxidized plasma membrane lipids is mainly caused by the activation of COX and LOX pathways in response to irradiation. Some of these lipid metabolites create a microenvironment favoring angiogenesis and cancer development. COX-2 is primarily responsible for cancer-associated inflammatory response, cancer cell malignancy, and radioresistance. For example, treatment with celecoxib, a selective COX-2 inhibitor, has been reported to augment radiosensitization through the activation of PTEN and the inhibition of AKT signaling [70,118], and treatment with zileuton (a 5-LOX inhibitor) had anti-angiogenic effects attributed to the suppression of vasculature formation through the downregulations of VEGF and MMP2 [119]. During normal ER stress response, PERK acts as a crucial cell survival factor through UPR signaling and eIF2α activation, while under conditions of excessive and chronic ER stress, such as those induced by irradiation, PERK is involved in the transcriptional upregulations of GADD34 and CHOP [97]. In glioblastoma cells, treatment with an eIF2α phosphatase inhibitor (e.g., Sal003) plus irradiation, blocked eIF2α dephosphorylation and prolonged eIF2α activity, and thus, promoted PERK activation and apoptosis [120]. Because of the cytoprotective effects of autophagy, its suppression might be expected to provide positive therapeutic outcomes by generating persistent oxidative stress and prolonging energy depletion during radiotherapy. Unfortunately, the prevention of autophagy by chloroquine or hydroxychloroquine administration resulted in no significant improvement in radiotherapeutic efficacy during clinical trials [121,122].

In tumor cells, the expressions of HIF1 and VEGF, induced by various cellular signals associated with hypoxia, ER stress, or autophagy, facilitate angiogenesis, which is closely correlated with tumor metastasis and radioresistance [123,124]. Along with HIF1 expression, hypoxia is a hallmark of solid tumors, and tumor cells in hypoxic regions may exhibit intrinsic radioresistance and contribute to disease aggressiveness and malignant development [125]. Many pre-clinical and clinical efforts have been made to overcome the effects of tumor hypoxia, and these include, the development of small molecules that target hypoxia-inducible signaling, the availability of oxygen in radiotherapy-targeted regions, and technologic improvements. Hyperthermia administered at from 39 to 45 °C is capable of enhancing oxygenation at tumor sites by increasing blood perfusion [126,127]. In addition to reoxygenation, hyperthermia can induce heat shock response, and involves the aggregation of denatured proteins and the activations of chaperon proteins like heat shock proteins (HSPs), the latter of which are associated with ER stress [128,129]. Damage induced by mild heat shock response can be recovered by the prompt activation of HSPs, such as HSP90, while hyperthermia combined with radiotherapy is likely to synergistically induce excessive stress and trigger apoptotic cell death [130,131]. Furthermore, hyperthermia might potentiate genomic instability and facilitate apoptotic signaling by enhancing radiation-induced DNA damage response. The formation of γH2AX/MDC1/53BP1 complexes, which are responsible for the repair of radiation-induced DSBs, is interrupted by heating prior to, or after, radiation, and enhances radiosensitivity [132]. In addition, the downregulation of BRCA, and the alterations in the localization of RAD51 might be induced by a combination of radiotherapy and hyperthermia, and thereby result in the inhibition of HR-dependent DNA repair and radiosensitization [133]. In a supportive study, that explored the hypothesis that NHEJ repair might be induced as a compensatory mechanism when HR repair is impeded by hyperthermia, treatment with a specific DNA-PK inhibitor for NHEJ inhibition enhanced the therapeutic efficacy of combinatorial radiotherapy and hyperthermia in tumor mouse models [134]. Based on its promising anti-cancer activity and radiosensitization effects, hyperthermia has been applied in clinical radiation oncology. Nevertheless, further investigations are required to validate the efficacy of combinatorial hyperthermia and radio-chemotherapy.

## 7. Conclusions

This review provides an overview of cellular stress response induced by irradiation, and includes considerations of the roles of ROS signaling, DNA damage response, membrane lipid peroxidation, mitochondrial damage, ER stress, and autophagy. Although, most cancer cells undergo cell death during radiotherapy, a small proportion of cells survive by activating DNA repair and survival signals, and as a result, acquire radioresistance (Table 1). In particular, cancer cells adapted to intratumoral hypoxia might be directed by HIF1 response, which includes metabolic reprogramming and survival signaling, and thus, acquire the ability to resist radiation-induced cytotoxic stress. Through the recovery of unfolded proteins and recycling of malfunctioned subcellular organelles, the activation of UPR and cytoprotective autophagy alleviate radiation-induced damage response, and thus, contribute to radioresistance. Although, tumor radioresistance remains a challenge, pre-clinical and clinical attempts in radiation oncology, such as combinatorial chemotherapeutic treatments with, or without, hyperthermia have improved the efficacy of radiotherapy. Further studies on the pharmacological applications of molecular radiosensitizers, in combination with, or without, hyperthermia, are required in different tumor microenvironments.

## Figures and Tables

**Table 1:** Cellular stress response associated with tumor radioresistance during radiotherapy.

Stress-Responsive Signaling for Radioresistance		Associated Molecules	Refs
**ROS stress response**	Upregulation of antioxidants	p53, SODs, glutathione peroxidase 1, sestrin	[24,25,26]
	Adaptation to hypoxia and inhibition of ROS production	HIF1, VEGF, PDK1	[33,34,35,36]
**DNA damage response**	Upregulation of DNA damage-sensing and repair proteins	ATM, γH2AX, DNA-PK, ATR, MDC1, BRCA1, BRCA2	[58,59,60]
**Subcellular organelle response**	Production of bioactive lipid metabolites	HNE (non-protein), COXs, LOXs	[63,70,71,72,73]
	Glycolytic reprogramming and mitochondrial malfunction	HIF1, PDK1	[81,82,83,84]
	Activation of UPR signaling	PERK, ATF4, ATF6, IRE1	[93,94,95,96,97,98]
**Autophagy**	Activation of cytoprotective autophagy	ATGs, ULK1, Beclin-1	[100,101,102,103,104,105]
